# C5aR1-positive neutrophils promote breast cancer glycolysis through WTAP-dependent m6A methylation of ENO1

**DOI:** 10.1038/s41419-021-04028-5

**Published:** 2021-07-26

**Authors:** Baochi Ou, Yuan Liu, Xiaowei Yang, Xiaojun Xu, Yunwen Yan, Jingjie Zhang

**Affiliations:** 1 Department of Breast Surgery, Department of General Surgery, The First Affiliated Hospital of Anhui Medical University, No. 218, Jixi Road, 230022 Hefei, Anhui China; 2 Department of General Surgery, Shanghai General Hospital, Shanghai Jiao Tong University, No. 85, Wujin Road, 200080 Shanghai, China

**Keywords:** Cancer microenvironment, Breast cancer, Oncogenesis

## Abstract

Neutrophils are significant compositions of solid tumors and exert distinct functions in different types of tumors. However, the precise role of neutrophils in the progression of breast cancer (BC) is presently unclear. In this study, by investigating the single-cell RNA sequencing data, we identify a new neutrophil subset, C5aR1-positive neutrophils, that correlates with tumor progression and poor survival for BC patients. Furthermore, it is discovered that C5aR1-positive neutrophils enhance BC cell glycolysis via upregulating ENO1 expression. Mechanically, C5aR1-positive neutrophil-secreted IL1β and TNFα cooperatively activate ERK1/2 signaling, which phosphorylates WTAP at serine341 and thereby stabilizes WTAP protein. The stabilization of WTAP further promotes RNA m6A methylation of ENO1, impacting the glycolytic activity of BC cells. Importantly, C5aR1-positive neutrophils also promote breast cancer growth in vivo, and this effect is abolished by WTAP silencing. In clinical BC samples, increased C5aR1-positive neutrophils correlate with elevated IL1β, TNFα, and ENO1 expression. A high co-expression of C5aR1-positive neutrophil gene signature and ENO1 predicts worse prognosis of BC patients compared with a low co-expression. Collectively, our study reveals a novel subset of C5aR1-positive neutrophils that induces breast cancer glycolysis via increasing ERK1/2-WTAP-dependent m6A methylation of ENO1. These findings support the potential for exploration of C5aR1-positive neutrophils as a therapeutic target in breast cancer.

## Introduction

Breast cancer (BC) becomes the most commonly diagnosed cancer worldwide, with 2.3 million new cases and over 690,000 deaths recorded in 2020 [1]. To date, the pathogenesis of BC is still unclear. The initiation and development of BC are not only governed by genetic alterations but also dependent on the interplay with surrounding microenvironment [2].

Tumor microenvironment comprises a variety of immune and nonimmune stromal cells, which are genetically stable and represent a promising therapeutic target [2]. Neutrophils constitute an abundant proportion in many types of malignancies, including BC [3]. They play a critical role in human inflammation and defense against microbial infections [4]. Recently, they are also recognized as a part of immune system to mediate tumor growth and progression. It is reported that parenchymal neutrophils are associated with poor prognosis of BC patients. The tumor-contacted neutrophils can induce BC metastasis via tissue inhibitor of matrix metalloprotease [5]. However, the precise role of neutrophils in carcinogenesis has been a matter of debate as they possess both pro- and anti-tumor properties [6–8]. Indeed, Hsu et al. find that immature low-density neutrophils are able to promote liver metastasis of BC, while high-density neutrophils suppress it [9]. Therefore, developing better strategies to identify different neutrophil subsets and their functions is required for precision treatment.

Cancer cells generally undergo aerobic glycolysis to meet the increased demands for biomass [10]. Aerobic glycolysis is characterized by much conversion of glucose into lactate regardless of oxygen availability. An enhanced aerobic glycolysis rate is a common metabolic feature in human malignancies and often correlates with tumor aggressiveness [11]. Emerging evidence has uncovered that aberrant metabolism in cancers is directed by genetic lesions, such as KRAS mutation and MYC amplification [12]. However, tumor metabolism is incredibly heterogenous and can also be affected by surrounding environments. For instance, cancer-associated fibroblasts can produce exosomes to boost glycolysis and reductive carboxylation of tumor cells [13]. Pancreatic stellate cells secret alanine to fuel the tricarboxylic acid (TCA) cycle of pancreatic cancer [14]. Hence, a better understanding of cancer energy metabolism is urgent for the development of new therapeutics.

In this work, we have shown that there exists a specific neutrophil subpopulation in the BC microenvironment: C5aR1-positive (C5aR1^+^) neutrophils. We found that C5aR1^+^ neutrophils promote glycolytic capacity of BC cells via upregulating the expression of Enolase 1 (ENO1). Moreover, C5aR1^+^ neutrophils-produced interleukin 1β (IL1β) and tumor necrosis factor α (TNFα) cooperatively activated extracellular signal-regulated kinase 1/2 (ERK1/2) signaling, thereby stabilizing WT1-associated protein (WTAP) to facilitate N6-methyladenosine (m6A)-dependent RNA methylation of ENO1. Notably, C5aR1^+^ neutrophils also promoted BC growth ability in an in vivo tumor model, which could be successfully abolished by WTAP knockdown.

## Results

### The critical role of C5aR1^+^ neutrophils in BC

**Fig. 1:**
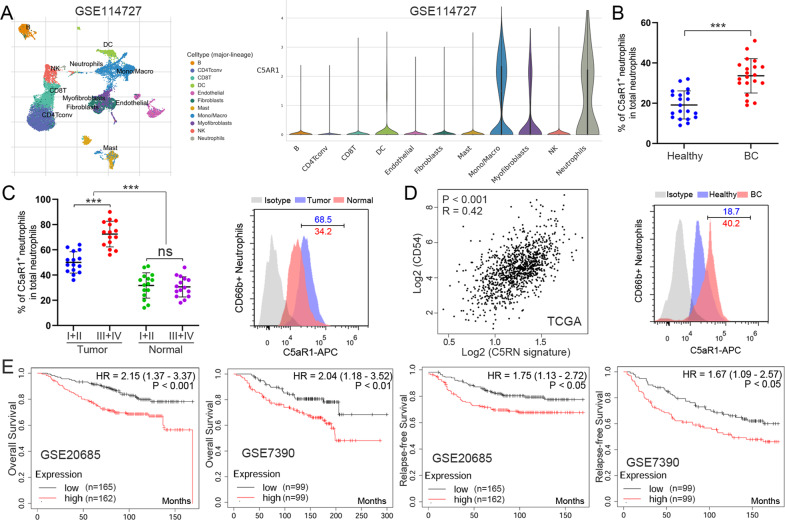
C5aR1^+^ neutrophils accumulate in breast cancer with disease progression and poor patient survival. **A** *t*-Distributed stochastic neighbor embedding plot of single-cell RNA-seq data from GSE114727. Identified clusters are represented by different colors (left). Violin plots showing expression of the indicated transcripts in each different cell type (right). **B** C5aR1^+^ neutrophil percentage in total CD66b^+^ neutrophils in peripheral blood from healthy donors or BC patients (top). Representative image of C5aR1 staining in CD66b^+^ neutrophils (bottom). **C** C5aR1^+^ neutrophil percentage in CD66b^+^ neutrophils in each tissue of patients with BC (left). Representative image of C5aR1 staining in CD66b^+^ neutrophils (bottom) (right). **D** The correlation between C5aR1^+^ neutrophil gene signature (CD66b, CD15, and C5aR1) and CD54 in human tumors was analyzed in TCGA. **E** Kaplan–Meier survival analysis showing overall survival and relapse-free survival based on the expression of C5aR1^+^ neutrophil gene signature in GSE20685 and GSE7390. ***P < 0.001.

To identify the specific subsets of neutrophils that play a role in BC, we first analyzed a Gene Expression Omnibus (GEO) dataset (GSE114727) containing single-cell RNA sequencing (RNA-seq) data from primary BC samples [15]. A list of differentially expressed genes was obtained within tumor-infiltrating neutrophils, compared to that extracted from matched normal tissues, blood, and lymph nodes (Table S1). We took a special interest in C5aR1, which is abundantly expressed by almost 77.2% of neutrophils in the BC environment (Fig. 1A and Table S1). To assess the potential role of C5aR1 on neutrophils in human BC, we determined C5aR1^+^ neutrophils (named C5RN) ratio within the total CD66b^+^ neutrophils in different tissues at various stages. Strikingly, patients with BC showed a higher C5RN percentage in peripheral blood than healthy donors (Fig. 1B). Within the patient cohort, tumors displayed a significantly higher C5RN percentage than normal tissues (Fig. 1C). Moreover, as the cancer progressed, we found that the percentage of C5RN significantly increased in each of the tested samples (Fig. 1C). To explore the clinical relevance of C5aR1^+^ neutrophils in BC, we generated a gene signature (CD66b, CD15, and C5aR1) to examine the abundance of C5RN in primary breast tumors. The correlation between C5RN gene signature and neutrophil activation marker CD54 was evaluated by using The Cancer Genome Atlas (TCGA). Intriguingly, C5RN gene signature and activation of neutrophils are correlated in human BC (Fig. 1D). Moreover, the high C5RN gene signature group had a worse overall survival and relapse-free survival than the low C5RN gene signature group, as shown in GSE20685 and GSE7390 (Fig. 1E). Taken together, these findings demonstrate that increased C5aR1^+^ neutrophils are associated with tumor progression and poor survival for BC patients.

### C5aR1^+^ neutrophils lead to enrichment of metabolites and genes associated with glycolysis in BC cells

**Fig. 2:**
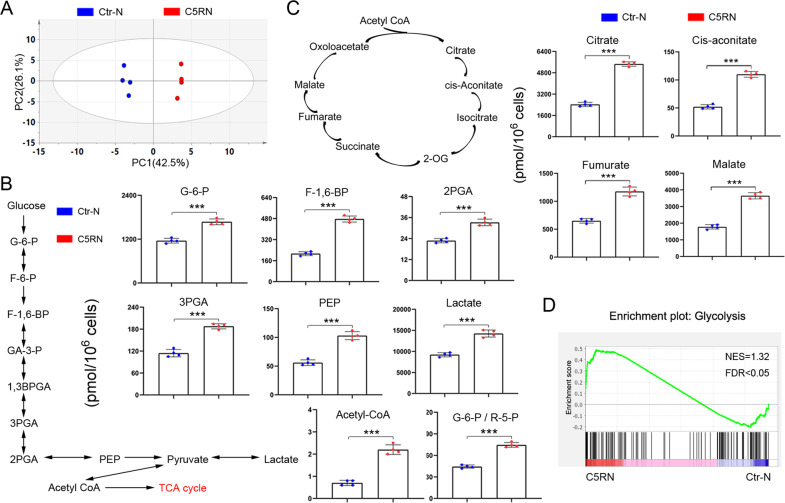
C5aR1^+^ neutrophils promote the glycolytic capacities of breast cancer cells. **A** Principal component analysis of metabolome profile comparing MCF-7 cells cultured with C5aR1^+^ neutrophils (C5RN) or C5aR1-negative neutrophils (Ctr-N). Data representative of four independent experiments. **B** Quantification of metabolic intermediates in glycolysis by metabolic profiling. Absolute values of metabolites are shown. **C** Quantification of metabolic intermediates in TCA cycle. Absolute values of metabolites are indicated. **D** Gene set enrichment analysis shows an enrichment of genes related to glycolysis in MCF-7^C5RN^ cells. Normalized enrichment score (NES) and false discovery rate (FDR) are indicated. Data represent the mean ± SD of at least three independent experiments. ***P < 0.001.

To examine the influence of C5aR1^+^ neutrophils on BC cells, we co-cultured C5RN with two cell lines, MCF-7 and MDA-MB-231 (tumor cells: neutrophils, 10:1), by using a non-contacting transwell system (Supplementary Fig. 1A). The CD66b^+^C5aR1^−^ neutrophils (named Ctr-N) were used as the control. During the experiments, we observed marked differences in the color of culture medium in C5RN-cultured cells relative to control group (Supplementary Fig. 1B). Generally, the phenol red in culture media gradually becomes yellow at lower pH values as a result of lactate generation. This finding leads us to assume whether C5RN play a role in modulating BC glucose metabolism. To this end, we performed metabolome profiling of C5RN-incubated MCF-7 cells (MCF-7^C5RN^). Principal component analysis suggested that the extractions were different between MCF-7^C5RN^ and control group (MCF-7^Ctr-N^, Fig. 2A). We found that a number of key metabolites involved in glycolysis and TCA cycle were enhanced in MCF-7^C5RN^ (Table S2). Specifically, MCF-7^C5RN^ cells exhibited an accumulation of glucose-6-phosphate (G6P), fructose 6-bisphosphate, 1,3-bisphosphoglyceric acid, 3-phosphoglyceric acid, 2-phosphoglyceric acid, phosphoenolpyruvic acid, lactate, and acetyl coenzyme A (Fig. 2B). Notably, the G6P/ribose 5-phosphate ratio was also increased (Fig. 2B), suggesting an enhanced glucose metabolism without diversion into the pentose phosphate pathway. In addition, levels of TCA cycle intermediates (citrate, *cis*-aconitate, fumarate, and malate) were significantly improved (Fig. 2C). This showed an increase in energy production via TCA cycle in MCF-7^C5RN^ cells. To further elucidate the effect of C5RN on BC cells, RNA-sequencing was conducted in the same cell lines. The mRNA profiling of MCF-7^C5RN^ revealed a significant enrichment of genes closely associated with glycolysis, as compared to control cells (Fig. 2D). These results indicate that C5aR1 may be a cell surface marker distinguishing the neutrophils, which can induce BC glycolysis.

### ENO1 knockout (KO) of BC cells reverses the glycolytic changes mediated by C5aR1^+^ neutrophils

**Fig. 3:**
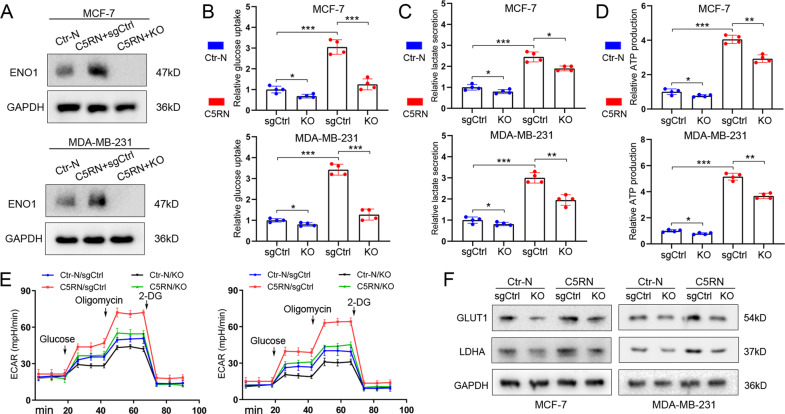
ENO1 KO reverses the C5RN-induced glycolysis of breast cancer cells. **A** Immunoblotting analysis of ENO1 in MCF-7 and MDA-MB-231 cells treated with C5aR1-negative neutrophils (Ctr-N) and C5aR1^+^ neutrophils (C5RN) with or without ENO1 knockout (KO). **B** ENO1 KO significantly alleviated the enhanced glucose uptake effect of C5RN on BC cells. **C** ENO1 KO significantly alleviated the enhanced lactate production effect of C5RN on BC cells. **D** ENO1 KO significantly alleviated the enhanced ATP production effect of C5RN on BC cells. **E** ENO1 KO significantly alleviated the enhanced ECAR effect of C5RN on BC cells. **F** Immunoblotting analysis of GLUT1 and LDHA in MCF-7 and MDA-MB-231 cells treated with C5RN with or without ENO1 KO. Data represent the mean ± SD of at least three independent experiments. *P < 0.05, **P < 0.01, ***P < 0.001.

Among the genes examined in the mRNA profiling, ENO1 was the most upregulated glycolysis-relevant gene in MCF-7^C5RN^ cells. To test whether ENO1 was essential for C5RN-induced glycolysis, we implemented CRISPR-Cas9 to KO ENO1 in MCF-7 and MDA-MB-231 cells. The CRISPR control single guide RNA (sgRNA) was used as the control group (sgCtrl). After confirming the KO effect (Fig. 3A), we found that C5aR1^+^ neutrophil-cultured tumor cells (MCF-7^C5RN^ and MDA-MB-231^C5RN^) exhibited an increase of glucose uptake, lactate secretion, and ATP production than the control group (Fig. 3B–D). This effect was significantly decreased upon ENO1 KO (Fig. 3B–D). To better determine the involvement of ENO1 in glycolysis-shifted metabolism in BC, we performed live monitoring to measure extracellular acidification rate (ECAR). Compared with the controls, C5RN incubation led to a significant increase in ECAR, a proxy for the rate of glycolysis and glycolytic capacity, while ENO1 KO abated this effect (Fig. 3E). In addition, ENO1 KO also alleviated the glycolytic capacity of tumor cells treated with control neutrophils (Fig. 3B–E). Meanwhile, we observed concomitant changes in the expression of key glycolytic genes like GLUT1 and LDHA (Fig. 3F and Supplementary Fig. 1C). Thus, these data indicate that ENO1 KO reverses the glycolysis mediated by C5aR1^+^ neutrophils in MCF-7 and MDA-MB-231 cells.

### ERK1/2-ENO1 signaling is responsible for C5aR1^+^ neutrophil-induced cancer glycolysis

**Fig. 4:**
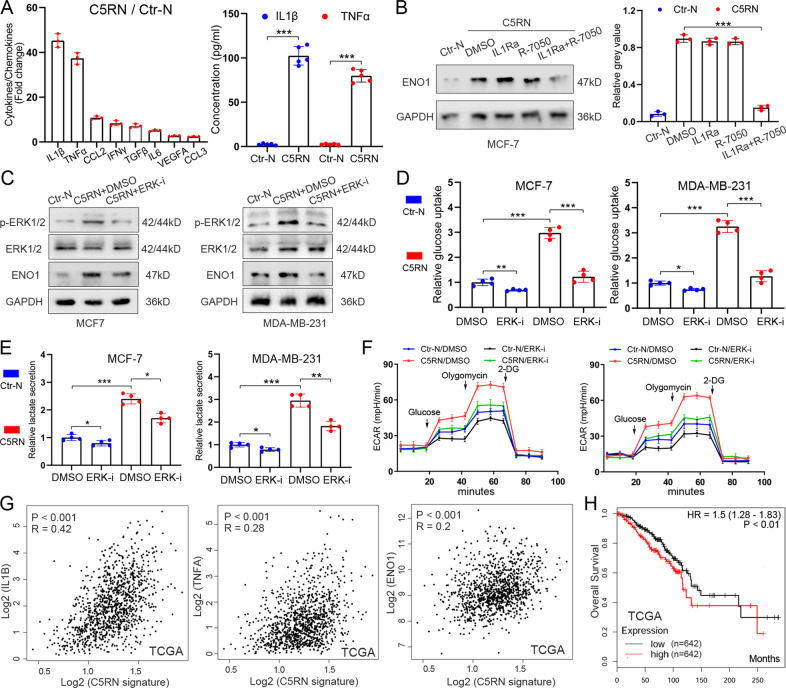
ERK1/2-ENO1 signaling is essential to C5aR1^+^ neutrophil-induced breast cancer cell glycolysis. **A** PCR array revealing a panel of significantly changed cytokine/chemokine mRNAs in C5aR1^+^ neutrophils (C5RN) relative to C5aR1-negative neutrophils (Ctr-N) (left). ELISA showing the expression of IL1β and TNFα in C5RN and Ctr-N (right). **B** Synergetic inhibition of IL1R and TNFR markedly abolished C5RN-induced ENO1 upregulation in BC cells. **C** Immunoblotting analysis of p-ERK1/2 (Thr202/Tyr204), ERK1/2, and ENO1 in MCF-7 and MDA-MB-231 cells with C5RN or C5RN plus ERK1/2 suppression (SCH772984). **D** Glucose uptake was determined in MCF-7 and MDA-MB-231 cells with C5RN or C5RN plus ERK1/2 inhibition (SCH772984). **E** Lactate production was examined in MCF-7 and MDA-MB-231 cells with C5RN or C5RN plus ERK1/2 inhibition (SCH772984). **F** Analysis of ECAR in MCF-7 and MDA-MB-231 cells with C5RN or C5RN plus ERK1/2 inhibition (SCH772984). **G** Scatter plot analysis revealing levels of L1β, TNFα, ENO1, and C5RN gene signature correlated in BC tissues. **H** Kaplan–Meier survival analysis showing overall survival based on the expression of C5RN gene signature and ENO1 in TCGA. Data represent the mean ± SD of at least three independent experiments. *P < 0.05, **P < 0.01, ***P < 0.001.

The neutrophils in tumor microenvironment play a crucial role in carcinogenesis via the secretion of various cytokines/chemokines. Thus, we propose that ENO1 upregulation in BC cells is triggered by C5aR1^+^ neutrophil-secreted cytokines and/or chemokines. Using a PCR array, we screened the mRNA expression of cytokines/chemokines and observed that several cytokines/chemokines were highly expressed in C5RN compared to Ctr-N (Fig. 4A). Among them, IL1β and TNFα were top two candidates and abundantly secreted by C5RN, as suggested by enzyme-linked immunosorbent assays (ELISAs) (Fig. 4A). However, blockade of IL1R (IL1Ra) or TNFR (R-7050) only slightly decreased the expression level of ENO1 induced by C5RN (Fig. 4B). When suppressing both IL1R and TNFR, we observed a significant downregulation of ENO1 in the cells (Fig. 4B). These results indicate that TNFα and IL1β may cooperatively but not solely promote ENO1 expression in BC cells. Considering that both TNFα and IL1β play pro-inflammatory roles by activating the signaling pathways within cells, we thus seek to determine the pathways responsible for ENO1 upregulation. We used inhibitors to target the most commonly aberrantly activated pathways and found that inhibition of ERK1/2 signaling with SCH772984 suppressed ENO1 protein expression in BC cells in a 24-h period (Fig. 4C and Supplementary Fig. 2A). Indeed, synergetic inhibition of TNFR and IL1R also suppressed the activation of ERK1/2 (Supplementary Fig. 2B). To further investigate the role of ERK1/2 in C5RN-induced glycolysis, we treated MCF-7^C5RN^ and MDA-MB-231^C5RN^ cells with SCH772984. The results showed that targeting ERK1/2 reduced glucose uptake (Fig. 4D), lactate production (Fig. 4E), and ECAR of MCF-7^C5RN^ and MDA-MB-231^C5RN^ (Fig. 4F), while ERK1/2 inhibition also suppressed the glycolysis of tumor cells treated with control neutrophils (Fig. 4D–F). Therefore, ERK1/2-ENO1 signaling is responsible for C5RN-induced glycolytic activity of BC cells.

Next, we evaluated the clinical relevance of C5RN gene signature, TNFα, IL1β, and ENO1 in BC. In patients with a high C5RN gene signature expression, the expression of TNFα, IL1β, and ENO1 tends to be low and vice versa (Fig. 4G), suggesting an indeed clinically important regulation. The expression of IL1β and TNFα was also positively correlated with ENO1 in BC tissues (Supplementary Fig. 2C). Furthermore, we found that a high C5RN gene signature and ENO1 co-expression predicts worse prognosis of BC patients compared with a low co-expression (Fig. 4H).

### C5aR1^+^ neutrophil-induced ERK1/2 activation controls m^6^A modification to upregulate ENO1 expression

**Fig. 5:**
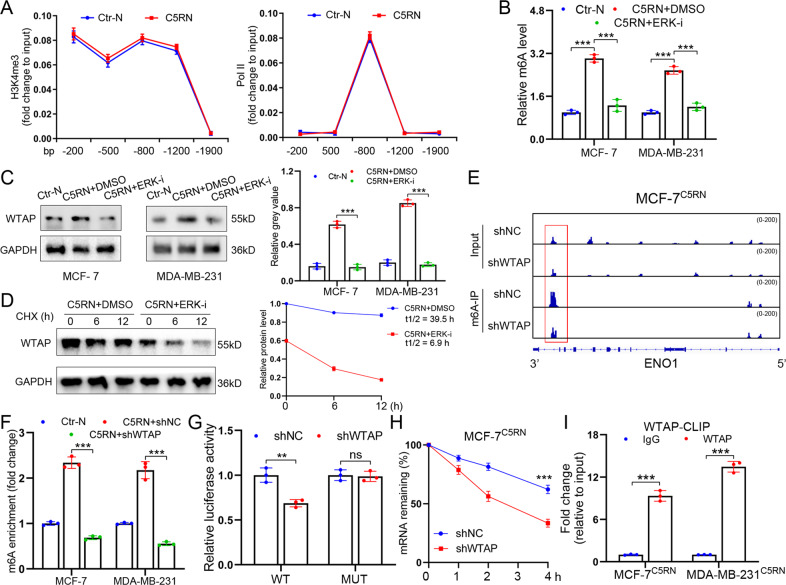
C5aR1^+^ neutrophils activate ERK1/2 signaling to sustain ENO1 expression through m6A modification. **A** H3K4me3 and Pol II abundance at gene promoters of ENO1 in MCF-7 stimulated by C5aR1^+^ neutrophils (C5RN) for 12 h or left unstimulated. **B** m6A levels of MCF-7^C5RN^ and MDA-MB-231^C5RN^ cells with or without ERK1/2 inhibition (SCH772984) were tested with the EpiQuik™ m6A RNA methylation quantification kit. **C** Immunoblotting and qPCR analysis for WTAP expression in MCF-7 and MDA-MB-231 treated with C5RN or C5RN plus ERK1/2 inhibition (SCH772984). **D** MCF-7 cells treated with C5RN or C5RN plus SCH772984 for 48 h, followed by cycloheximide (CHX, 10 mg/mL) for 0–12 h. Lysates were used to measure the protein levels of WTAP. The relative fold is indicated and plotted in the right panel. **E** m6A peaks were enriched at ENO1 mRNA from m6A-RIP sequencing data of MCF-7^C5RN^ cells. **F** m6A enrichment of ENO1 in MCF-7^C5RN^ and MDA-MB-231^C5RN^ cells with or without WTAP silencing. **G** Dual luciferase reporter assays showed the effect of WTAP on ENO1 reporters with either WT or MUT binding sites. **H** After treatment with actinomycin D (Act-D) for the indicated times, the mature mRNA levels of ENO1 were checked in MCF-7^C5RN^ cells with or without WTAP silencing. **I** CLIP-qPCR showing the association of ENO1 transcripts with WTAP in MCF-7^C5RN^ and MDA-MB-231^C5RN^ cells. Data represent the mean ± SD of at least three independent experiments. **P < 0.01, ***P < 0.001, Ctr-N C5aR1-negative neutrophils.

We then sought to determine whether C5aR1^+^ neutrophil-induced ERK1/2 signaling upregulated ENO1 expression on a transcriptional level. With chromatin immunoprecipitation (ChIP) experiments, we observed that the abundance of chromatin activation mark H3K4me3 and the occupancy of DNA polymerase II at the promoter regions of ENO1 were not influenced by C5aR1^+^ neutrophil stimulation (Fig. 5A). Thus, we wondered whether or not C5RN stimulation facilitated ENO1 expression via post-transcriptional regulation of RNA stability. m6A is the first established and most common internal modification of RNA that widely affects RNA stability. As such, we tested whether ENO1 upregulation in BC cells was mediated in an m6A-relevant manner. Interestingly, the global m6A level in MCF-7^C5RN^ was boosted compared with that in the control cells, which was abolished by ERK1/2 inactivation (Fig. 5B). It is known that methyltransferase or demethylase mainly control the m6A level. To study whether C5RN stimulation induces m6A level via RNA methyltransferases or demethylases, the levels of METTL3, METTL14, WTAP, FTO, and ALKBH5 were examined in MCF-7^C5RN^. We found that C5RN cultivation promoted the expression of WTAP protein without affecting its mRNA levels, whereas inhibition of ERK1/2 signaling by SCH772984 reduced WTAP protein expression in cancer cells (Fig. 5C and Supplementary Fig. 2D). Using cycloheximide (CHX) chase assay, we also demonstrated that inhibition of ERK1/2 triggered WTAP degradation (Fig. 5D). Strikingly, the expression of other proteins exhibited no significant change (Supplementary Fig. 3A, B). These results indicate that ERK1/2 plays a vital role in WTAP protein stabilization in C5RN-cultured tumor cells.

To investigate whether WTAP controls m6A modification to promote ENO1 expression, we determined the existence of m6A modification on ENO1 by m6A RNA immunoprecipitation (m6A-RIP) sequencing. Through analyzing the m6A-RIP sequencing data, we detected several significant m6A peaks that were reduced by WTAP silencing in the 3′-untranslated region (3′UTR) of ENO1 mRNA (Fig. 5E). Indeed, knockdown of WTAP decreased m6A-methylated ENO1 mRNAs in MCF-7^C5RN^ and MDA-MB-231^C5RN^, which was consistent with the m6A-dependent function of WTAP (Fig. 5F). We then constructed luciferase reporters containing 3′UTR of ENO1 (pGL3-ENO1-WT) or control mutant (pGL3-ENO1-MUT). As expected, silencing WTAP reduced the luciferase activity of the pGL3-ENO1-WT vector, while mutation in the m6A sites abolished the inhibition (Fig. 5G). Additionally, knockdown of WTAP decreased the mRNA stability of ENO1 in MCF-7^C5RN^ and MDA-MB-231^C5RN^ cells (Fig. 5H and Supplementary Fig. 3C). Considering that WTAP is essential for the function of METTL3/METTL14 complex, we then inhibited METTL3 or METTL14 using small-interfering RNAs. The data revealed that silencing either METTL3 or METTL14 could also reduce the stability of ENO1 mRNA (Supplementary Fig. 3D). To confirm the direct interaction between ENO1 mRNAs and WTAP protein, we further performed cross-linking and RNA immunoprecipitation (CLIP) quantitative real-time PCR (qPCR) assay. As shown in Fig. 5I, ENO1 mRNAs can interact with WTAP in both MCF-7^C5RN^ and MDA-MB-231^C5RN^, suggesting that ENO1 are direct targets of WTAP. Moreover, WTAP silencing reduced mRNA levels of ENO1 in C5RN-cultured tumor cells (Supplementary Fig. 3E). Thus, C5aR1^+^ neutrophils activate ERK1/2-WTAP signaling to sustain ENO1 expression in BC cells.

### WTAP phosphorylation at S341 by ERK1/2 stabilizes WTAP to promote BC cell glycolysis

**Fig. 6:**
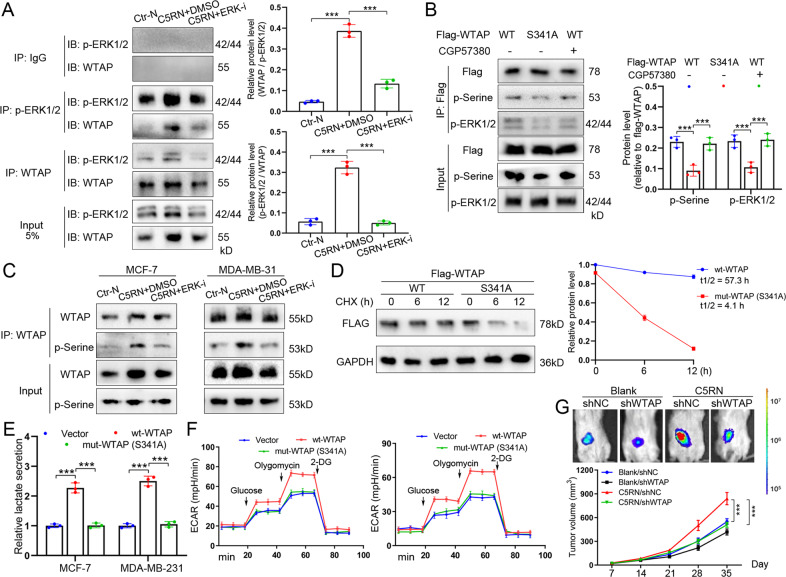
WTAP phosphorylation at serine 341 by ERK1/2 stabilizes WTAP to promote breast cancer cell glycolysis. **A** Co-IP of WTAP with p-ERK1/2 (Thr202/Tyr204) in whole-cell extracts from MCF-7^C5RN^ cells with or without ERK1/2 inhibition (left). Quantitative analysis of co-interaction between WTAP and p-ERK1/2 (right). **B** Co-IP of p-ERK1/2 (Thr202/Tyr204) with WT or S341A mutated Flag-WTAP in whole-cell extracts from MCF-7^C5RN^ cells treated with or without 10 μM CGP57380. The phosphoserine (p-Serine) of immunoprecipitated Flag-WTAP were detected using pan-phosphoserine antibody. **C** Immunoblotting of phosphoserine (p-Serine) of immunoprecipitated WTAP in MCF-7^C5RN^ and MDA-MB-231^C5RN^ cells with or without ERK1/2 inhibition. **D** MCF-7^C5RN^ cells were cultured by CHX (10 mg/mL) for 0–12 h. Lysates were used to measure the protein levels of FLAG. The relative fold is indicated and plotted in the right panel. **E** Lactate production was examined in MCF-7 and MDA-MB-231 cells transfected with vector, wild-type, or mutant WTAP (S341A). **F** Analysis of ECAR in MCF-7 and MDA-MB-231 cells transfected with vector, wild-type, or mutant WTAP (S341A). **G** Effects of breast cancer cells with C5RN culture and shWTAP transfection on tumor growth using mice model. The volumes are measured every 7 days. Data represent the mean ± SD of at least three independent experiments. ***P < 0.001, Ctr-N C5aR1-negative neutrophils.

To investigate how ERK1/2 signaling maintains WTAP expression, we performed reciprocal immunoprecipitation assays in BC cells. Interestingly, WTAP was able to associate with active ERK1/2 in MCF-7^C5RN^, which was further alleviated by ERK1/2 suppression (Fig. 6A). Moreover, we conducted in vitro kinase assays using recombinant ERK2 and purified WTAP. It was shown that WTAP was phosphorylated by ERK2, whereas the inhibitor SCH772984 abrogated phosphorylation (Supplementary Fig. 4A). We then determined whether ERK1/2 phosphorylated WTAP since the ERK1/2 inhibitor SCH772984 inhibited WTAP expression. According to GPS 5.0 (http://gps.biocuckoo.cn/), several serine and threonine sites in WTAP may be phosphorylated by ERK1/2 (Supplementary Fig. 4B). Among these sites, S306 and S341 phosphorylations have been documented in a previous research [16]. However, only S341 of WTAP is conserved across different mammalian species (Supplementary Fig. 4C), suggesting that it is most likely the ERK phosphorylation site. To examine this hypothesis, we constructed S341A mutant of WTAP and observed that S341A mutation largely abrogated the association of ERK1/2 with WTAP (Fig. 6B). Using the phosphoserine antibodies, we confirmed that S341 of WTAP2 was phosphorylated in MCF-7^C5RN^ cells (Fig. 6B). Notably, the S341A mutant displayed a clear signal with p-Serine antibody, suggesting that another residue of WTAP might also be phosphorylated by ERK1/2. Additionally, C5RN stimulation increased serine phosphorylation of WTAP in MCF-7 and MDA-MB-231, while ERK1/2 suppression decreased the phosphorylation (Fig. 6C). On the other hand, inhibition of the ERK1/2 downstream kinase MNK1 by CGP57380 failed to affect WTAP phosphorylation at S341 site (Fig. 6B), suggesting WTAP as a direct target of ERK1/2.

To determine the function of the WTAP phosphorylation, we first measured the stability of exogenous FLAG-tagged WTAP. S341A mutant displayed an accelerated degradation compared with the wild type (Fig. 6D). In addition, the m6A level is markedly lower in S341A mutant relative to the wild-type WTAP (Supplementary Fig. 4D). Next, S341A mutant was expressed to a similar amount as wild-type WTAP (judged by the similar protein levels, Supplementary Fig. 4E) and its glycolytic effect was tested. Intriguingly, S341A mutant exerted minimal effects on the promotion of lactate production (Fig. 6E) and ECAR (Fig. 6F) of BC cells compared with wild-type WTAP. To further study whether WTAP stabilized by C5RN can affect BC growth in vivo, we injected the cells transfected with shWTAP or shNC, with or without C5RN (10:1), into nude mice. It was observed that C5RN significantly contributed to the growth of breast tumors, while WTAP inhibition largely attenuated this effect (Fig. 6G).

**Fig. 7:**
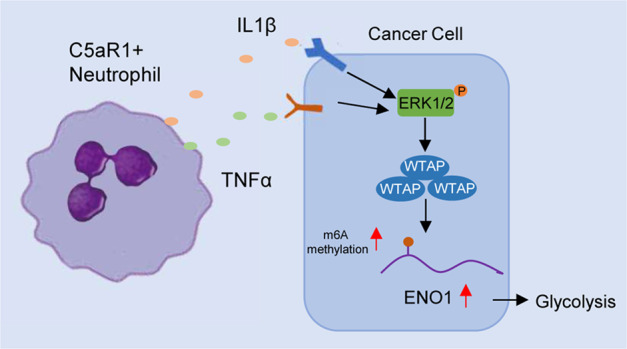
Proposed model for C5aR1+ neutrophils in reprogramming breast cancer cell glycolysis. C5aR1-positive neutrophils-secreted IL1β and TNFα cooperatively activate ERK1/2 signaling, which phosphorylates WTAP at serine341 and thereby stabilizes WTAP protein. The stabilization of WTAP further promotes RNA m6A methylation of ENO1, impacting the glycolytic activity of BC cells.

Collectively, the schematic diagram of this study is shown in Fig. 7.

## Discussion

The cross-talk between cancer cells and surrounding stroma extensively affects tumor progression and patient prognosis [2]. As the most abundant leukocytes, neutrophils are an essential component of tumor microenvironment and play a role in carcinogenesis. They can display anti-tumor abilities such as functioning as antigen-presenting cells and direct tumor cell killing [17, 18]. Conversely, neutrophils have also been shown to promote metastasis by secreting cytokines or by undergoing the NETosis [5, 19]. These findings suggest the existence of phenotypic heterogeneity and functional versatility within neutrophils and that targeting all neutrophils is not an appropriate approach for anticancer therapies.

C5aR1 is the specific receptor of the complement C5a. C5a–C5aR1 axis has been implicated in inflammatory disease progression and tumorigenesis. It is demonstrated that C5a exerts many functions through the activation of C5aR1 on immune cells, such as macrophages, neutrophils, and dendritic cells [20]. Notably, C5aR1 signaling can stimulate neutrophil mobilization and suppress their apoptosis [21, 22]. Apart from its role as immune modulators, C5aR1 expression in tumor cells is involved in the activation of various signals that promote cancer development [23, 24]. However, the function of C5aR1 on neutrophils during cancer progression remains unknown. Here, by analyzing BC single-cell RNA-seq data, we found that C5aR1 was highly expressed in tumor-infiltrating neutrophils relative to that from normal tissues and blood. Moreover, increased C5aR1^+^ neutrophils were linked to tumor progression and poor survival for BC patients. Similarly, a recent study identifies a unique neutrophil subclone that lacks P2RX1 and participates in immunosuppression and cancer metastasis [25]. These studies indicate that the characterization of different neutrophil subsets based on specific molecules can guide the development of neutrophil-targeting precision therapies. In this study, the gene of CD66b, CD15 and C5aR1 was used to represent the signature of C5aR1 neutrophils. This representation is a little limited and not perfect. A more precise gene signature for C5aR1^+^ neutrophils is needed in future research.

Cancer cells with reinforced aerobic glycolysis are a pivotal hallmark for tumorigenesis [10]. Although recent studies have identified some intrinsic factors that modulate BC cell glycolysis, the metabolic switching of BC cells in response to inflammatory microenvironment has not been fully explored. In this study, by using metabolome profiling and RNA-seq, we revealed that C5RN enhanced the glycolytic capacity of BC cells. Furthermore, ENO1 KO largely rescued the metabolic rewiring induced by C5RN incubation, supporting that C5RN could facilitate BC cell glycolysis via the enzyme ENO1. It is well established that ENO1 is a metabolic enzyme participating in the synthesis of pyruvate. The upregulation of ENO1 has been reported in BC and is associated with tumor aggression [26]. ENO1 could be modulated by a variety of mechanisms, such as hypoxic stress, c-MYC, and ERK1/2 [27]. Herein our study indicates that ENO1 of tumor cells can also be regulated by neutrophils in the BC microenvironment. This reveals a novel coupling of BC cells with neighboring non-neoplastic cells through ENO1-dependent glucose metabolism.

Neutrophils are able to secret immunoregulatory cytokines and chemokines to influence cancer cell biology and tumor microenvironment. In this study, we focused on the metabolic effect of neutrophils on BC cells and identified IL1β and TNFα as a vital bridge that connected C5RN and tumor cells. Subsequent investigations demonstrated that these two cytokines cooperatively activated ERK1/2 signaling to promote ENO1-dependent glycolytic traits of cancer cells. Although it is established that ERK1/2 signaling regulates cell survival and proliferation in certain tumors [28], this study shows that ERK1/2 activation enhances BC cell glycolysis and plays a role in C5RN-induced effects. Moreover, we confirmed in BC public database that levels of C5RN gene signature, IL1β, TNFα, and ENO1 were positively correlated. A high C5aR1^+^ neutrophils gene signature and ENO1 co-expression predicts worse prognosis of patients compared with a low co-expression. However, whether other stromal cells like tumor-associated macrophages and cancer-associated fibroblasts could affect ERK1/2 activation of BC cells remains undefined.

Accumulating evidence has uncovered many molecular mechanisms involved in gene expression, including transcriptional and post-transcriptional regulation. Here, after excluding the possibility of ENO1 mediated by C5RN on a transcriptional level, we observed a direct post-transcriptional regulation of ENO1 by WTAP-dependent m6A methylation. m6A is the most prevalent internal modification in eukaryotic mRNA. m6A methylation is mediated by a complex of three proteins: METTL3, METL14, and WTAP, known as “writers” [29]. Among them, WTAP is responsible for the recruitment of METTL3 and METTL14 to nuclear speckles. There is evidence that WTAP participates in tumorigenesis and cancer progression [30]. Recent study also documents that WTAP accelerates glucose metabolism of gastric cancer [31]. Here our findings highlight the significance of WTAP-mediated m6A methylation in C5aR1^+^ neutrophil-induced tumor glycolysis, suggesting a critical role of WTAP in tumor metabolism.

Currently, the role of methyltransferase WTAP has been established, but the mechanism of how it is regulated remains unclear. It is reported that METTL3 can mediate WTAP protein homeostasis. In the lack of METTL3, WTAP is not sufficient to boost cell proliferation [32]. Recently, Han et al. shows that a PIWI-interacting RNA is able to upregulate WTAP in lymphoma [30]. In this study, we provide evidence that ERK1/2 phosphorylates WTAP at serine341, thus stabilizing WTAP protein to promote BC cell glycolysis. Our findings are consistent with a previous study, which suggests that ERK can phosphorylate both METTL3 and WTAP, facilitating USP5-mediated stabilization of m6A methyltransferase complex in embryonic stem cells [16].

Collectively, we identify a specific neutrophil subpopulation, C5aR1^+^ neutrophils, that correlates with tumor progression and poor survival of BC patients. Furthermore, C5aR1^+^ neutrophils promote BC cell glycolytic capacity through the ERK1/2-WTAP-ENO1 signaling. Mechanically, WTAP is phosphorylated at S341 and thus stabilized by ERK1/2 in the context of C5aR1^+^ neutrophils. These findings highlight the potential for C5aR1^+^ neutrophils and the WTAP–ENO1 network as therapeutic targets for BC.

## Materials and methods

### Patients and neutrophil isolation

Tumor tissues and peripheral blood were obtained from BC patients who underwent surgical resection at Shanghai General Hospital and the First Hospital of Anhui Medical University between 2019 and 2020. None of these patients had received chemotherapy or radiotherapy before surgery. The study was approved by the Ethics Committee of Shanghai General Hospital and the First Hospital of Anhui Medical University. Written informed consent was obtained from each subject. Fresh BC tissues were cut into pieces and digested in RPMI 1640 medium with 20% fetal bovine serum (FBS), 0.002% DNase I (Roche), and 0.05% collagenase IV (Sigma-Aldrich) at 37 °C for 30 min. The dissociated cells were filtered through a 70-μm mesh and stained with antihuman CD66b and/or antihuman C5aR1 antibodies. Then the single-cell suspension was sorted by fluorescence activating cell sorter (FACS; BD Biosciences). Additionally, neutrophils from peripheral blood of patients or healthy donors were isolated by staining CD66b and/or C5aR1 with FACS. The sorted cells were not used unless their purity was determined to be >85%.

### Cells and reagents

BC cell lines (MCF-7, MDA-MB-231) and HEK293T cells were obtained from Chinese Academy of Sciences and grown in Dulbecco’s Modified Eagle Medium (DMEM). Tumor cells and neutrophils were co-cultured (tumor cells: neutrophils, 10:1) in DMEM with 10% FBS. We changed the conditioned medium daily to remove apoptotic neutrophils and added fresh neutrophils. Before performing functional experiments or examining gene expression, conditioned medium was changed to remove suspended or apoptotic neutrophils. All cell lines were authenticated by short tandem repeat profiling and tested for mycoplasma. For inhibitor treatment, the indicated cells were cultured with the inhibitor of ERK1/2 (SCH772984), TNFR (R-7050), CGP57380, and IL1R (anakinra, named IL1Ra) in suggested concentration for 24 h.

### Lentiviral transduction and transient transfection

ENO1 was knocked out by infecting cells with viruses produced using LentiCRISPR V2 plasmids [33], which contained ENO1-specific sgRNA (AGG TCC TAC CTT GCT AAC CA) or control sgRNA (GCG AGG TAT TCG GCT CCG CG). Lentiviral vectors expressing non-targeting control short hairpin RNA (shRNA; SCH002), and shRNA targeting WTAP (TRCN0000001078) were purchased from Sigma-Aldrich. The lentiviral vectors were co-transfected with packaging vectors psPAX2 and pMD2.G (Addgene) for lentivirus production. To establish stable cell lines, cancer cells were transduced by lentiviruses with polybrene (8 µg/mL). After 72 h of transfection, target cell lines were selected in puromycin-containing media. Ten days following selection, cells were harvested for subsequent experiments. Transient transfections were performed using Lipofectamine2000 following the manufacturer’s protocols. WTAP with N-terminal DYK tag expression plasmid and small-interfering RNAs were synthesized from GenePharma (China). WTAP S341A mutant was generated using the QuikChange II XL Site-Directed Mutagenesis Kit (Agilent) following the instructions.

### qPCR, PCR array, RNA-seq, and ELISA

Total RNA was extracted using TRIzol (Invitrogen) and cDNA was synthesized by PrimeScript RT Master Mix (Takara). qPCR was performed with ABI 7900HT Real-Time PCR system (Applied Biosystems) with the listed primers in Table S3. We conducted the human Cytokine & Chemokine RT^2^ profiler PCR array according to the manufacturer’s instructions. This PCR array contains 84 key genes involved in interleukins, cytokines, growth factors, and so on. RNA-seq was performed as a service at Majorbio (Shanghai, China). We conducted pathway analyses on the differentially expressed genes using Gene Set Enrichment Analysis. Moreover, the conditioned medium collected from C5aR1^+^ neutrophils or controls was analyzed with IL1β and TNFα ELISA kits from R&D Systems.

### Metabolite analysis

After removing the media, cells were washed with 0.9% NaCI and quenched with 100% cold methanol, followed by the addition of isovolumetric water. We scraped the cells thoroughly and transferred into an Eppendorf tube containing 400 μL chloroform. Next, cells were vibrated at 1400 rpm for 20 min, followed by centrifugation at 16,100 × *g* for 5 min. Samples were then dried at a low temperature under vacuum to avoid metabolite degradation. The extracted intracellular metabolites were analyzed by gas chromatography and time-of-flight mass spectrometry, provided by BioMaker (China) as a service. We determined glycolytic capacity of cancer cells by using the Glucose Uptake Colorimetric Assay Kit (Biovision), Lactate Colorimetric Assay Kit (Biovision), and ATP Assay Kit (Promega), according to the manufacturers’ instructions. Glucose-induced ECAR was monitored by the Seahorse XF24 Flux Analyzer as previously described [34]. Cells were first seeded in a XF24 well plate and cultured overnight. Then the cells were washed with Seahorse buffer, followed by sequentially injecting 10 mM glucose, 1 µM oligomycin, and 80 mM 2-deoxyglucose. ECAR measurements were normalized to the number of cells per well.

### Immunoblotting and co-immunoprecipitation (Co-IP)

Immunoblotting was performed as previously described [35]. Antibodies against ENO1 (ab155102, dilution 1:1000), WTAP (ab195380, dilution 1:1000), phosphoserine (ab9332, dilution 1:100), FLAG (ab1162, dilution 1:2000), and GAPDH (ab9484, dilution 1:5000) were purchased from Abcam. Antibodies against GLUT1 (12939, dilution 1:1000), LDHA (3582, dilution 1:1000), ERK1/2 (4695, dilution 1:1000), and p-ERK1/2 (Thr202/Tyr204,4370, dilution 1:2000) were purchased from Cell Signaling Technology. Co-IP was performed as previously documented [36]. Cells were lysed in immunoprecipitation buffer and then were incubated with the indicated primary antibodies or an isotype IgG antibody (Santa Cruz). The incubation was shaken on a rotating shaker at 4 °C overnight. Then the protein complexes were immunoprecipitated by protein A/G agarose (Santa Cruz). We collected the protein complexes and added 2× sample loading buffer to boil with them. The supernatant was then analyzed by immunoblotting.

### In vitro kinase

Cells transfected with Flag-tagged WTAP were lysed in IP lysis buffer and immunoprecipitated with anti-FLAG M2 beads. The precipitated WTAP proteins were eluted by 3× Flag peptide (150 μg/mL) diluted in kinase buffer (20 mM Tris–HCl, pH 7.5, 10 mM MgCl_2_, 5 mM dithiothreitol). Recombinant active ERK2 (E1283) was purchased from Sigma-Aldrich. In all, 200 ng purified Flag-WTAP was incubated in 25 μL kinase buffer containing 0.5 mM ATP at 30 °C for 30 min, in the presence of 100 ng ERK2. The reaction was stopped by adding the sample buffer, followed by immunoblotting analysis.

### Animal models

Four-week-old male BALB/c nude mice were used in this study (seven randomly allocated for each group). All mice were housed in a specific pathogen-free environment. A total of 1 × 10^6^ luciferase-labeled BC cells transfected with shWTAP or shNC were injected subcutaneously with or without C5aR1^+^ neutrophils (tumor cells:neutrophils, 10:1). Neutrophils were injected into the tumor twice a week for 3 weeks, beginning at day 14 after inoculation. After 5 weeks, mice were anesthetized and intraperitoneally injected with D-luciferin (100 mg/kg) and imaged 10 min after injection, using the IVIS Illumina System (Caliper Life Sciences). Xenograft sizes were measured every week and calculated as: *V* = (Width^2^ × Length)/2. All animal experiments were conducted with the approval of the Animal Ethics Committee (Anhui Medical University).

### Chromatin immunoprecipitation

ChIP was performed using the ChIP Assay Kit (Millipore) according to the manufacturer’s instructions. Gene enrichment was quantified using qPCR and presented as fold change to input. The ChIP antibodies against H3K4me3 (9751) and Pol II subunit I Rpb1 (2629) were purchased from Cell Signaling Technology. Primers for amplification of ENO1 gene promoters are listed in Table S3.

### m6A quantification

The mRNA was first purified by using two rounds of the Dynabeads mRNA Purification Kit (Thermo Scientific). The global m6A levels in mRNA were calculated with the EpiQuik m6A RNA Methylation Quantification Kit (Epigentek, Farmingdale, NY) according to the manufacturer’s protocol. In all, 200 ng poly-A-purified RNA of each sample was used for analysis.

### m6A-RIP qPCR and sequencing

m6A-RIP qPCR and sequencing were performed as previously reported [37, 38]. Total RNA was extracted by TRIzol from the indicated cells and sheared into approximately 100 nt fragments with RNA fragmentation reagents (Invitrogen). Then fragmented RNA was incubated with m6A antibody for RIP according to the protocol of Magna methylated RNA Immune-precipitation m6A Kit (Merck Millipore, USA). The methylated RNA was purified and further determined by qPCR with the primers listed in Table S3 or by m6A sequencing. For sequencing, purified RNA from m6A-RIP were used for library construction with the NEBNext Ultra RNA library Prep Kit for Illumina (NEB, USA). Sequencing was performed on Illumina HiSeq 2000 according to the manufacturer’s instructions. Sequencing reads were aligned to the human genome GRCh38/hg38, and the m6A peaks were tested by magnetic cell sorting as described.

### Luciferase assay

The fragments of ENO1-3’UTR containing wild-type m6A motifs as well as mutant m6A sites (m6A was replaced by T) were obtained from GenePharma (Shanghai, China). The wild-type and mutant ENO1-3’UTR fragments were inserted into the downstream of pGL3-basic firefly luciferase vector. Tumor cells were co-transfected with 500 ng wild-type or mutant ENO1-3’UTR and 100 ng pRL-TK plasmid (Renilla luciferase). After 48 h of incubation, the relative luciferase activity was detected using the Dual-Luciferase Reporter Assay System (Promega, USA).

### mRNA and protein stability

RNA stability of tumor cells was conducted by incubating cells with actinomycin D (Santa Cruz) at 5 µM. Cells were collected at the indicated times and total RNA was isolated for qPCR analysis. Half-life of ENO1 mRNA was calculated using ln2/slope and normalized to GAPDH according to the described method [39]. To detect protein stability, cells were treated with CHX (50 mg/mL) and harvested at 0, 6, and 12 h.

### Cross-linking and RNA immunoprecipitation qPCR

The nuclear extracts of tumor cells were first isolated and sonicated. We conjugated 1 mg of WTAP antibody or isotype IgG to Protein A/G Magnetic Beads (Santa Cruz) by incubating them at 4 °C for 4 h. The mixture was then washed and incubated with prepared nuclear contents in RIP buffer at 4 °C overnight. After 3 times washing with RIP buffer, beads were suspended in phosphate-buffered saline with DNA digestion, followed by incubation with Proteinase K (Thermo Fisher) at 37 °C for 15 min. Input and co-immunoprecipitated RNAs were recovered by TRIzol (Invitrogen) and used for subsequent qPCR analyses.

### Public dataset analysis

We determined the expression and correlation of C5RN gene signature, ENO1, IL1β, and TNF-α in BC by using the data from TCGA. The prognostic values of C5RN gene signature and ENO1 were examined in GEO or TCGA. Moreover, we performed single-cell RNA-seq analysis based on the TISCH database [40].

### Statistical analysis

Data were presented as mean ± SD of at least three independent tests. Student’s *t* test was used to compare quantitative data between groups. Survival rates were calculated by the Kaplan–Meier method, and differences were analyzed by log-rank test. Correlations between parameters were assessed using the Pearson correlation analysis. Values of *P* < 0.05 were considered significant.

## Supplementary information

Supplementary Table

Supplementary Figure legends

Figure S1

Figure S2

Figure S3

Figure S4

## Data Availability

The dataset supporting the conclusions of this article is included within the article. The m6A-RIP sequencing data are deposited in The Sequence Read Archive with the accession number PRJNA735577.
