# Supplementary material for: C5aR1-positive neutrophils promote breast cancer glycolysis through WTAP-dependent m6A methylation of ENO1
Source: Cell Death Dis. 2021 Jul 26;12(8):737. doi: 10.1038/s41419-021-04028-5 (PMC8313695; doi:10.1038/s41419-021-04028-5)
Supplement: Supplementary file 1 — Supplementary Table [file 41419_2021_4028_MOESM1_ESM.docx]

**Table S1.** Lists of differentially expressed genes in tumor-infiltrating neutrophils of breast cancer.

| Cell type (malignancy) | Cell type | Gene | log2FC | Percentage (%) | Adjusted p-value |
| --- | --- | --- | --- | --- | --- |
| Immune cells | Neutrophils | S100A9 | 2.6 | 98.7 | 1.47E-40 |
| Immune cells | Neutrophils | S100A8 | 2.14 | 91.1 | 6.98E-30 |
| Immune cells | Neutrophils | CSF3R | 1.71 | 94.9 | 7.35E-29 |
| Immune cells | Neutrophils | IFITM2 | 1.79 | 98.7 | 7.36E-29 |
| Immune cells | Neutrophils | CXCR2 | 1.86 | 87.3 | 6.82E-26 |
| Immune cells | Neutrophils | FPR1 | 1.77 | 91.1 | 2.19E-25 |
| Immune cells | Neutrophils | FCGR3B | 2.08 | 86.1 | 6.72E-25 |
| Immune cells | Neutrophils | NEAT1 | 1.37 | 97.5 | 1.33E-22 |
| Immune cells | Neutrophils | ATG16L2 | 1.28 | 84.8 | 3.30E-19 |
| Immune cells | Neutrophils | BASP1 | 1.55 | 91.1 | 4.78E-19 |
| Immune cells | Neutrophils | SLC25A37 | 1.59 | 89.9 | 3.94E-18 |
| Immune cells | Neutrophils | SPI1 | 1.33 | 87.3 | 5.69E-17 |
| Immune cells | Neutrophils | MYO1F | 1.15 | 87.3 | 5.69E-17 |
| Immune cells | Neutrophils | SRGN | 1.45 | 92.4 | 5.69E-17 |
| Immune cells | Neutrophils | S100A11 | 0.85 | 96.2 | 1.55E-15 |
| Immune cells | Neutrophils | ADGRE5 | 0.98 | 91.1 | 1.73E-15 |
| Immune cells | Neutrophils | G0S2 | 1.59 | 82.3 | 4.31E-15 |
| Immune cells | Neutrophils | HSH2D | 1.21 | 77.2 | 1.09E-14 |
| Immune cells | Neutrophils | CYP4F3 | 0.75 | 72.2 | 5.82E-14 |
| Immune cells | Neutrophils | S100P | 1.5 | 74.7 | 3.60E-13 |
| Immune cells | Neutrophils | ALOX5AP | 1.24 | 86.1 | 5.10E-13 |
| Immune cells | Neutrophils | RP5-1171I10.5 | 0.71 | 82.3 | 5.87E-13 |
| Immune cells | Neutrophils | FGR | 0.83 | 82.3 | 7.24E-13 |
| Immune cells | Neutrophils | RGS2 | 0.92 | 83.5 | 1.10E-12 |
| Immune cells | Neutrophils | MMP25 | 1.14 | 79.7 | 4.40E-12 |
| Immune cells | Neutrophils | PGLYRP1 | 0.55 | 65.8 | 1.26E-11 |
| Immune cells | Neutrophils | LILRB3 | 0.92 | 79.7 | 1.26E-11 |
| Immune cells | Neutrophils | GNLY | 0.87 | 79.7 | 1.34E-11 |
| Immune cells | Neutrophils | AMICA1 | 1.05 | 84.8 | 1.63E-11 |
| Immune cells | Neutrophils | ITGAX | 0.92 | 81 | 1.78E-11 |
| Immune cells | Neutrophils | NAMPT | 1.07 | 79.7 | 1.98E-11 |
| Immune cells | Neutrophils | SAT1 | 0.74 | 88.6 | 3.63E-11 |
| Immune cells | Neutrophils | CXCR1 | 0.87 | 73.4 | 3.63E-11 |
| Immune cells | Neutrophils | KLRD1 | 1.07 | 78.5 | 4.13E-11 |
| Immune cells | Neutrophils | DUSP1 | 0.93 | 94.9 | 5.82E-11 |
| Immune cells | Neutrophils | TYROBP | 0.62 | 86.1 | 9.74E-11 |
| Immune cells | Neutrophils | CEBPB | 0.86 | 87.3 | 1.15E-10 |
| Immune cells | Neutrophils | AQP9 | 1.19 | 77.2 | 2.23E-10 |
| Immune cells | Neutrophils | ACTB | 0.44 | 100 | 3.70E-10 |
| Immune cells | Neutrophils | NKG7 | 0.48 | 75.9 | 5.18E-10 |
| Immune cells | Neutrophils | FTH1 | 0.65 | 97.5 | 6.70E-10 |
| Immune cells | Neutrophils | BCL6 | 1.11 | 82.3 | 7.12E-10 |
| Immune cells | Neutrophils | FCGR2A | 0.74 | 81 | 7.91E-10 |
| Immune cells | Neutrophils | ADGRG3 | 0.91 | 74.7 | 9.27E-10 |
| Immune cells | Neutrophils | SOD2 | 0.88 | 81 | 9.58E-10 |
| Immune cells | Neutrophils | FFAR2 | 1.07 | 75.9 | 1.28E-09 |
| Immune cells | Neutrophils | CST7 | 1.34 | 81 | 1.28E-09 |
| Immune cells | Neutrophils | IFIT3 | 0.86 | 73.4 | 3.71E-09 |
| Immune cells | Neutrophils | CCL4 | 1.96 | 75.9 | 5.21E-09 |
| Immune cells | Neutrophils | RAB37 | 0.39 | 72.2 | 5.60E-09 |
| Immune cells | Neutrophils | CCL5 | 1.07 | 78.5 | 1.03E-08 |
| Immune cells | Neutrophils | EFHD2 | 0.86 | 83.5 | 1.58E-08 |
| Immune cells | Neutrophils | SELL | 0.85 | 89.9 | 1.74E-08 |
| Immune cells | Neutrophils | RPS12 | -0.67 | 98.7 | 1.90E-08 |
| Immune cells | Neutrophils | S100A12 | 0.89 | 70.9 | 2.17E-08 |
| Immune cells | Neutrophils | IRF1 | 1.09 | 78.5 | 2.88E-08 |
| Immune cells | Neutrophils | LRRC25 | 0.75 | 74.7 | 3.29E-08 |
| Immune cells | Neutrophils | PI3 | 0.65 | 59.5 | 3.45E-08 |
| **Immune cells** | **Neutrophils** | **C5AR1** | **1.19** | **77.2** | **5.52E-08** |
| Immune cells | Neutrophils | NCF2 | 0.98 | 74.7 | 5.52E-08 |
| Immune cells | Neutrophils | MXD1 | 0.97 | 73.4 | 6.04E-08 |
| Immune cells | Neutrophils | LST1 | 0.63 | 82.3 | 8.08E-08 |
| Immune cells | Neutrophils | CXCR4 | 0.72 | 84.8 | 1.00E-07 |
| Immune cells | Neutrophils | KLRG1 | 0.39 | 69.6 | 1.00E-07 |
| Immune cells | Neutrophils | FTL | 0.46 | 97.5 | 1.04E-07 |
| Immune cells | Neutrophils | STX3 | 0.56 | 73.4 | 1.11E-07 |
| Immune cells | Neutrophils | MX2 | 0.91 | 79.7 | 1.82E-07 |
| Immune cells | Neutrophils | P2RY13 | 0.32 | 69.6 | 1.82E-07 |
| Immune cells | Neutrophils | CTSW | 0.59 | 73.4 | 1.86E-07 |
| Immune cells | Neutrophils | ALPL | 0.75 | 73.4 | 2.23E-07 |
| Immune cells | Neutrophils | TNFAIP2 | 0.63 | 75.9 | 2.28E-07 |
| Immune cells | Neutrophils | IL2RB | 0.79 | 75.9 | 2.53E-07 |
| Immune cells | Neutrophils | MBOAT2 | 0.31 | 65.8 | 2.98E-07 |
| Immune cells | Neutrophils | AIF1 | 0.52 | 78.5 | 3.78E-07 |
| Immune cells | Neutrophils | SERPINA1 | 0.62 | 78.5 | 4.48E-07 |
| Immune cells | Neutrophils | SLC11A1 | 1.05 | 77.2 | 4.86E-07 |
| Immune cells | Neutrophils | APOBEC3A | 0.8 | 74.7 | 5.15E-07 |
| Immune cells | Neutrophils | PDLIM7 | 0.7 | 70.9 | 6.31E-07 |
| Immune cells | Neutrophils | LRG1 | 0.39 | 70.9 | 7.29E-07 |
| Immune cells | Neutrophils | TREM1 | 0.86 | 72.2 | 7.29E-07 |
| Immune cells | Neutrophils | VNN2 | 0.82 | 72.2 | 7.38E-07 |
| Immune cells | Neutrophils | CCL4L2 | 0.34 | 68.4 | 8.65E-07 |
| Immune cells | Neutrophils | TAGLN2 | 0.7 | 89.9 | 9.21E-07 |
| Immune cells | Neutrophils | C10ORF54 | 0.72 | 86.1 | 1.45E-06 |
| Immune cells | Neutrophils | SECTM1 | 0.66 | 74.7 | 1.46E-06 |
| Immune cells | Neutrophils | HMGB2 | 0.69 | 79.7 | 1.47E-06 |
| Immune cells | Neutrophils | MNDA | 1.14 | 75.9 | 2.00E-06 |
| Immune cells | Neutrophils | GZMA | 0.7 | 73.4 | 2.67E-06 |
| Immune cells | Neutrophils | MEFV | 0.38 | 67.1 | 3.50E-06 |
| Immune cells | Neutrophils | TCN1 | 0.25 | 60.8 | 4.06E-06 |
| Immune cells | Neutrophils | FCER1G | 0.55 | 78.5 | 4.21E-06 |
| Immune cells | Neutrophils | LYN | 0.67 | 79.7 | 4.36E-06 |
| Immune cells | Neutrophils | CLEC4E | 0.99 | 70.9 | 4.92E-06 |
| Immune cells | Neutrophils | PADI4 | 0.56 | 68.4 | 5.00E-06 |
| Immune cells | Neutrophils | TRGC1 | 0.41 | 67.1 | 5.21E-06 |
| Immune cells | Neutrophils | GLIPR2 | 0.81 | 79.7 | 5.35E-06 |
| Immune cells | Neutrophils | TBX21 | 0.37 | 69.6 | 6.91E-06 |
| Immune cells | Neutrophils | LINC01272 | 0.72 | 69.6 | 7.11E-06 |
| Immune cells | Neutrophils | RP4-635A23.6 | 0.93 | 74.7 | 7.11E-06 |
| Immune cells | Neutrophils | HCK | 0.45 | 74.7 | 8.09E-06 |

**Table S2.** Lists of differentially expressed metabolites in MCF-7 cells cultured with C5aR1^+^ neutrophils or control cells.

| Metabolites | Control* (n=4) | TANs* (n=4) | *P* value |
| --- | --- | --- | --- |
| Glucose-6-phosphate | 1157.56±65.53 | 1680.62±74.15 | <0.001 |
| Fructose-1,6-phosphate | 213.45±14.82 | 476.78±22.91 | <0.001 |
| 1,3-bisphosphoglyceric acid | 50.13±3.18 | 83.83±3.34 | <0.001 |
| 3-phosphoglyceric acid | 114.54±7.86 | 187.82±6.95 | <0.001 |
| 2-phosphoglyceric acid | 22.74±1.26 | 33.24±1.95 | <0.001 |
| Phosphoenolpyruvic acid | 56.13±4.92 | 103.27±6.82 | <0.001 |
| L-lactate | 9227.52±487.93 | 14295.14±765.26 | <0.001 |
| Acetyl coenzyme A | 0.73±0.15 | 2.20±0.22 | <0.001 |
| Citrate | 2425.15±154.38 | 5455.42±164.21 | <0.001 |
| Cis-aconitate | 52.03±3.91 | 109.95±5.10 | <0.001 |
| Fumarate | 649.57±40.33 | 1175.24±78.53 | <0.001 |
| Malate | 1778.54±131.99 | 3864.75±187.53 | <0.001 |
| ATP | 6518.41±336.65 | 12238.37±294.39 | <0.001 |
| ADP | 815.26±58.02 | 1457.35±58.52 | <0.001 |
| Isoleucine | 128.26±13.09 | 108.16±7，41 | 0.038 |
| L-alanine | 17.75±2.99 | 11.25±2.50 | 0.016 |
| L-serine | 315.75±23.18 | 263.25±11.30 | 0.007 |
| L-threonine | 37.12±4.69 | 46.25±4.50 | 0.029 |
| L-valine | 4.93±0.92 | 7.08±0.74 | 0.011 |
| Phenylalanine | 16.52±1.29 | 12.74±1.71 | 0.013 |
| Tyrosine | 50.04±3.65 | 40.75±5.32 | 0.028 |
| Valine | 81.13±5.48 | 100.25±8.26 | 0.008 |

*Average ± SD

**Table S3.** Primers used in this study.

| Gene | Sequences (5’---3’) | |
| --- | --- | --- |
| ENO1 | sense | AAAGCTGGTGCCGTTGAGAA |
|  | antisense | GGTTGTGGTAAACCTCTGCTC |
| WTAP | sense | CTTCCCAAGAAGGTTCGATTGA |
|  | antisense | TCAGACTCTCTTAGGCCAGTTAC |
| METTL3 | sense | TTGTCTCCAACCTTCCGTAGT |
|  | antisense | CCAGATCAGAGAGGTGGTGTAG |
| METTL14 | sense | GAACACAGAGCTTAAATCCCCA |
|  | antisense | TGTCAGCTAAACCTACATCCCTG |
| FTO | sense | ACTTGGCTCCCTTATCTGACC |
|  | antisense | TGTGCAGTGTGAGAAAGGCTT |
| ALKBH5 | sense | CGGCGAAGGCTACACTTACG |
|  | antisense | CCACCAGCTTTTGGATCACCA |
| ENO1 promotor | sense | CTCCAACTCCTTCCGTATT |
|  | antisense | GACCCTGTCCCTTTCTCC |
| 3'UTR of ENO1 | sense | TGGGATAGACGCAGACTTAG |
|  | antisense | GAGGTAGGAGAATCGCTTGA |
