# Supplementary material for: C5aR1-positive neutrophils promote breast cancer glycolysis through WTAP-dependent m6A methylation of ENO1
Source: Cell Death Dis. 2021 Jul 26;12(8):737. doi: 10.1038/s41419-021-04028-5 (PMC8313695; doi:10.1038/s41419-021-04028-5)
Supplement: Supplementary file 2 — Supplementary Figure legends [file 41419_2021_4028_MOESM2_ESM.docx]

**Supplementary Figure legends**

**Supplementary Figure 1 A** Schematic illustration of the *in vitro* coculture system. **B** Representative bright-field images of culture media color in MCF-7 and MDA-MB-231 cells. **C** Immunoblotting analysis for GLUT1 and LDHA expression in MCF-7 and MDA-MB-231 cells treated with C5RN or C5RN plus ENO1 KO. Densitometry represents the expression of the proteins relative to GAPDH. ****P* < 0.001.

**Supplementary Figure 2 A** Immunoblotting analysis of p-ERK1/2 (Thr202/Tyr204), ERK1/2 and ENO1 in MCF-7 and MDA-MB-231 cells with C5RN or C5RN plus ERK1/2 suppression (SCH772984). **B** Immunoblotting analysis of p-ERK1/2 (Thr202/Tyr204) in MCF-7 cells treated with C5RN with or without IL1Ra plus R-7050. **C** Scatter plot analysis revealing levels of IL1β, TNFα and ENO1 correlated in BC tissues. **D** qPCR analysis of WTAP in MCF-7 and MDA-MB-231 cells with or without C5RN co-culture. ****P* < 0.001, ns, not significant.

**Supplementary Figure 3 A** Immunoblotting analysis of METTL3, METTL4, FTO and ALKBH5 in MCF-7 cells treated with C5RN or Ctr-N (C5aR1-negative neutrophils). Densitometry represents the expression of the proteins relative to GAPDH. **B** qPCR analysis of METTL3, METTL4, FTO and ALKBH5 in MCF-7 cells treated with C5RN or Ctr-N. **C** After treatment with actinomycin D (Act-D) for the indicated times, the mature mRNA levels of ENO1 were checked in MDA-MB-231^C5RN^ cells with or without WTAP silencing. **D** The mature mRNA levels of ENO1 were checked in cells treated with Act-D for the indicated times, with the knockdown of METTL3 or METTL14. **E** qPCR analysis of ENO1 in MCF-7^C5RN^ and MDA-MB-231^C5RN^ cells with or without WTAP inhibition. ****P* < 0.001, ns, not significant.

**Supplementary Figure 4 A** In vitro kinase assay of ERK2 with purifed Flag-WTAP as a substrate. The reaction samples were immunoblotted for the serine phosphorylation level of WTAP. **B** Predicted phosphorylation sites of WTAP by GPS 5.0 software. **C** Sequence alignment of WTAP from multiple species based on predicted ERK1/2 phosphorylation sites. **D** The m6A quantification of MCF-7 cells transfected with Flag-WTAP wild-type or S341A mutant. **E** Immunoblotting analysis of WTAP in MCF-7 cells transfected with Flag-WTAP wild-type or mutant expression plasmids (S341A). ns, not significant.
