# Supplementary figures and images for: C5aR1-positive neutrophils promote breast cancer glycolysis through WTAP-dependent m6A methylation of ENO1
Source: Cell Death Dis. 2021 Jul 26;12(8):737. doi: 10.1038/s41419-021-04028-5 (PMC8313695; doi:10.1038/s41419-021-04028-5)

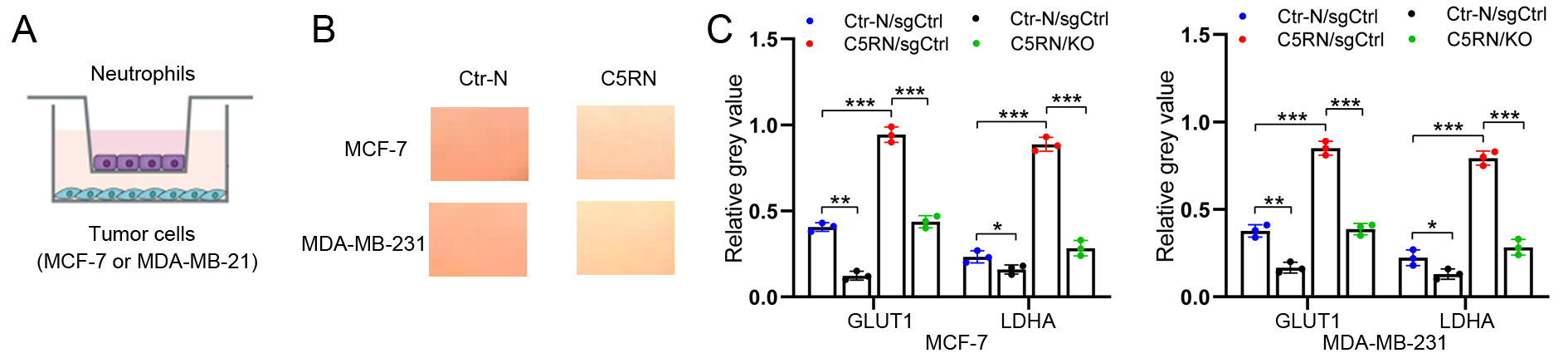

Supplement: Supplementary file 3 — Figure S1 [file 41419_2021_4028_MOESM3_ESM.tif]

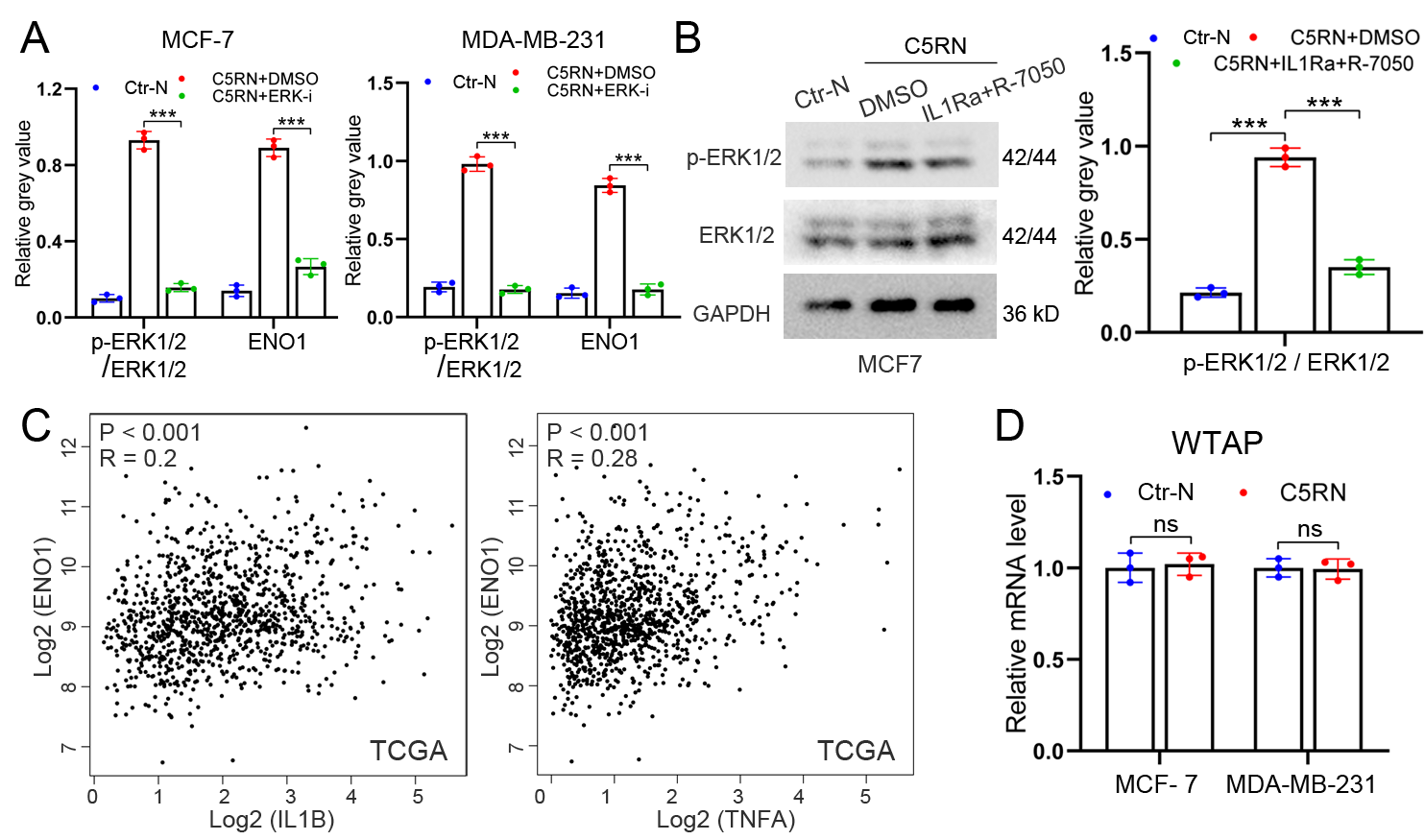

Supplement: Supplementary file 4 — Figure S2 [file 41419_2021_4028_MOESM4_ESM.tif]

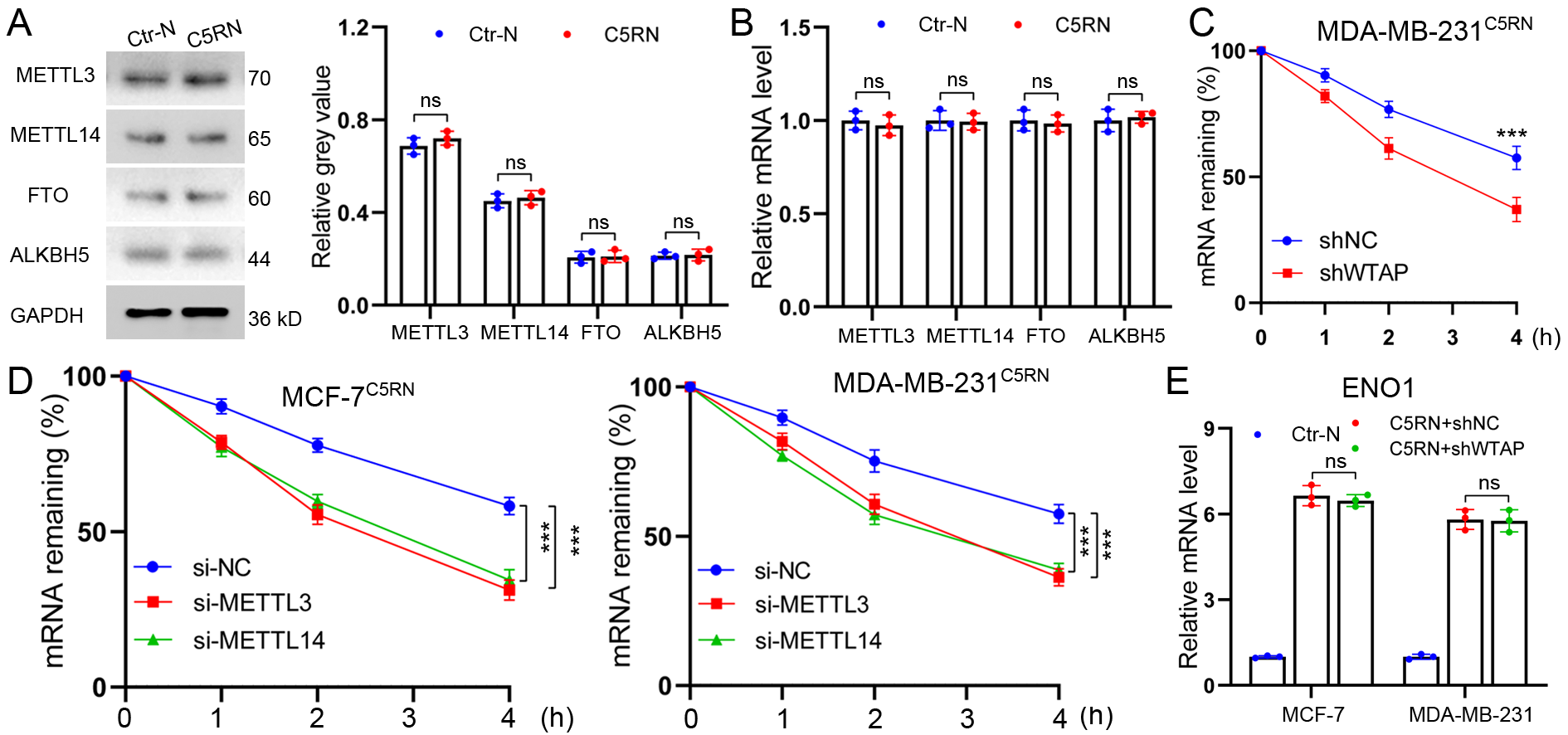

Supplement: Supplementary file 5 — Figure S3 [file 41419_2021_4028_MOESM5_ESM.tif]

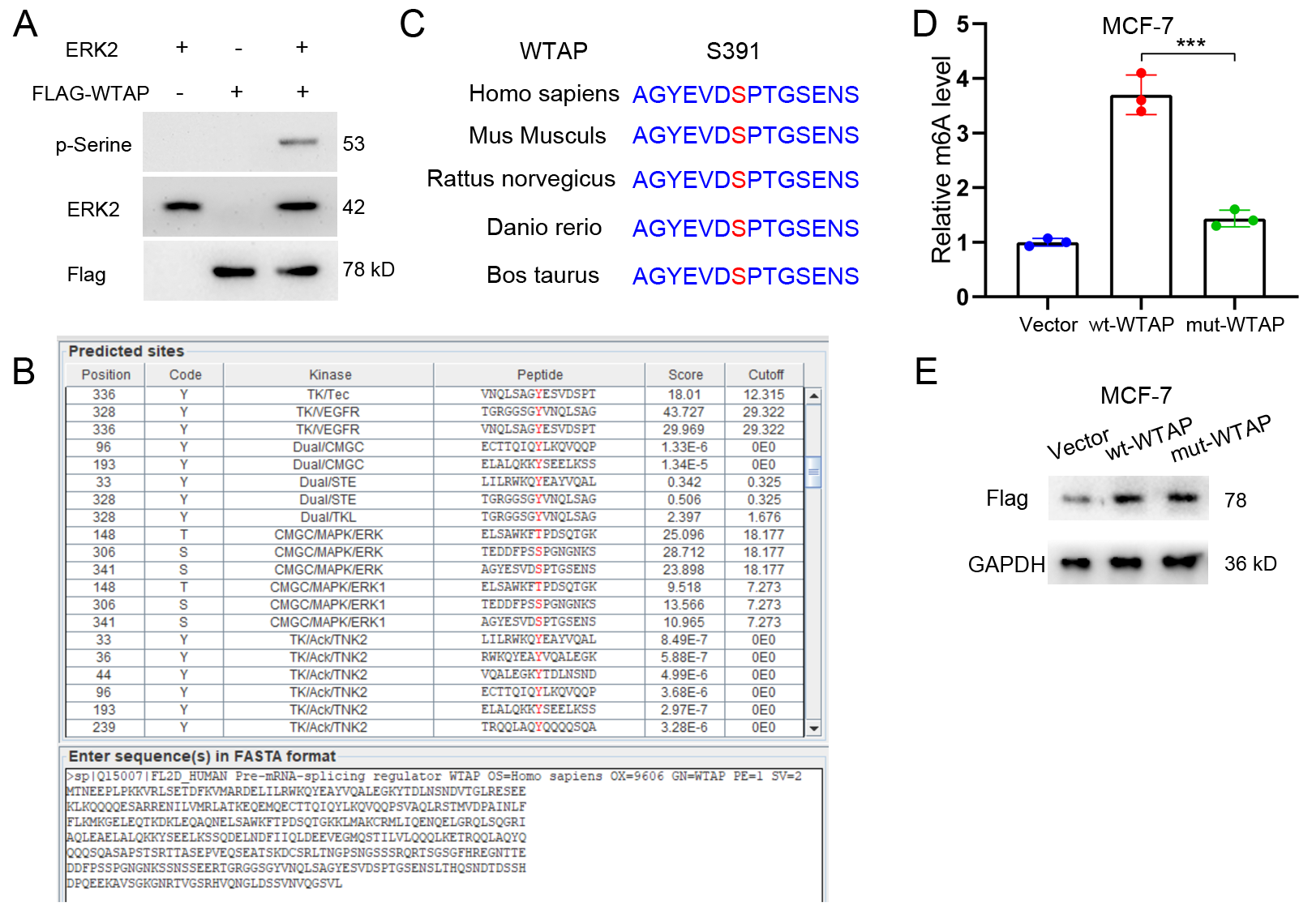

Supplement: Supplementary file 6 — Figure S4 [file 41419_2021_4028_MOESM6_ESM.tif]
